# Diagnostic value of T_1_- and T_2_-weighted 3-Tesla MRI for postmortem detection and age stage classification of myocardial infarction

**DOI:** 10.1007/s12024-023-00592-8

**Published:** 2023-03-02

**Authors:** Dominic Gascho, Alexandre von Allmen, Anna Landsmann, Tobias Hünermund, Carlo Tappero, Michael J. Thali, Eva Deininger-Czermak

**Affiliations:** 1 Department of Forensic Medicine and Imaging, Zurich Institute of Forensic Medicine, University of Zurich, Zurich, Switzerland; 2 Institute of Diagnostic and Interventional Radiology, University Hospital Zurich, Zurich, Switzerland; 3 Department of Radiology, Hôpital Fribourgeois, Fribourg, Switzerland

**Keywords:** Myocardial infarction, Forensic medicine, Postmortem changes, Magnetic resonance imaging

## Abstract

The aims of this study are to retrospectively evaluate the diagnostic value of T_1_- and T_2_-weighted 3-T magnetic resonance imaging (MRI) for postmortem detection of myocardial infarction (MI) in terms of sensitivity and specificity and to compare the MRI appearance of the infarct area with age stages. Postmortem MRI examinations (*n* = 88) were retrospectively reviewed for the presence or absence of MI by two raters blinded to the autopsy results. The sensitivity and specificity were calculated using the autopsy results as the gold standard. A third rater, who was not blinded to the autopsy findings, reviewed all cases in which MI was detected at autopsy for MRI appearance (hypointensity, isointensity, hyperintensity) of the infarct area and the surrounding zone. Age stages (peracute, acute, subacute, chronic) were assigned based on the literature and compared with the age stages reported in the autopsy reports. The interrater reliability between the two raters was substantial (*κ* = 0.78). Sensitivity was 52.94% (both raters). Specificity was 85.19% and 92.59%. In 34 decedents, autopsy identified an MI (peracute: *n* = 7, acute: *n* = 25, chronic: *n* = 2). Of 25 MI classified as acute at autopsy, MRI classified peracute in four cases and subacute in nine cases. In two cases, MRI suggested peracute MI, which was not detected at autopsy. MRI could help to classify the age stage and may indicate the area for sampling for further microscopic examination. However, the low sensitivity requires further additional MRI techniques to increase the diagnostic value.

**Supplementary Information:**

The online version contains supplementary material available at 10.1007/s12024-023-00592-8.

## Introduction

Myocardial infarction (MI) is a common natural cause of death and is frequently detected during forensic medical examinations as part of the process of ruling out a non-natural cause of death. The macroscopic appearance of MI and the tissue changes of the myocardium usually allow detection of MI and classification of their age stage into recent (acute) or older (subacute and chronic) at autopsy. Additional histologic examination usually allows accurate classification of age stage, except in very recent (peracute) MI. Detecting such a peracute infarct is difficult with conventional autopsy and histology, even with additional immunohistochemically analysis [1]. With the increasing advent of noninvasive postmortem imaging, the question arises to what extent imaging can detect MI and classify the age stage.

Previous studies have shown that frequently used postmortem computed tomography (CT) is useful to measure the size of the heart on CT images to estimate heart weight, visualize hemopericardium and calcified plaques and valves, and detect and locate cardiovascular devices, or, in the context of CT angiography, to examine the coronary arteries for the extent and location of narrowing and obstruction [2]. However, the sensitivity of postmortem CT for detecting areas of infarction in the myocardium is considered insufficient. For postmortem detection of MI, another imaging modality has been evaluated that is sensitive to pathologic changes in tissue: postmortem cardiac magnetic resonance imaging (MRI). For instance, the difference in relaxation times and density of hydrogen protons in fluids compared to hydrogen protons in tissue allows contrast between edematous and non-edematous myocardial tissue. In addition, the ischemic death of myocardial tissue and the accompanying change in relaxation times and proton density in the infarct area allow this affected tissue to be distinguished from unaffected myocardial tissue. Jackowski et al. [3] studied the different appearances of MI on postmortem MRI in 76 decedents with MI and inferred different ages from the dark (hypointense) and bright (hyperintense) areas detected. The authors concluded that T_2_-weighted images can show the infarct area (central zone) dark in the peracute phase, which is then surrounded by a brighter zone in the acute phase (myocardial edema). In the subacute phase, the central zone becomes brighter and the surrounding zone loses brightness and becomes indistinguishable from unaffected tissue (isointense). In the chronic phase, the central zone finally becomes dark again. On the T_1_-weighted images, the central zone is isointense in the peracute, acute, and subacute age stage until it finally appears dark in the chronic phase. Myocardial edema in the acute phase is shown dark. These correlations between the age stage of MI and the MRI appearance of MI on T_1_-weighted and T_2_-weighted images are consistent with the findings of previous small case studies [4, 5].

While these studies provided promising results for postmortem detection of MI on postmortem MRI, the study conducted in 2018 by Wagensveld et al. [6] on the diagnostic value of T_1_-weighted and T_2_-weighted imaging for MI detection in a hospital setting showed less promising results in terms of sensitivity. The examinations were performed with a 1.5-T scanner using an 8-channel array coil. In a study group of 99 decedents, 34 (peracute to) acute MI and 40 (subacute to) chronic MI were detected by conventional autopsy. The specificities of postmortem MRI for the detection of acute and chronic MI were high at 92% (CI_95%_: 83–97%) and 100% (CI_95%_: 94–100%), respectively. However, the sensitivities were relatively low at 50% (CI_95%_: 32–68%) for acute MI and at 35% (CI_95%_: 21–52%) for chronic MI. The authors attributed the low sensitivity to the short examination time available and the use of a 1.5-T scanner. More time and a higher magnetic field strength (*B*_0_) of the scanner have a positive effect on the base signal-to-noise ratio (SNR). On the one hand, a four times longer scan time (*t*) would allow doubling the SNR (at the same voxel size (Δ*V*)), and on the other hand, the base SNR of a 3-T scanner is about twice as large as the base SNR of a 1.5-T scanner [7]:

$$\Delta V\cdot \sqrt{t} \sim { SNR \sim B}_{0}$$

In addition, the SNR is further improved by the configuration and number of channels of the MRI receiver coil. Therefore, the question arises whether the sensitivity for MI detection can be improved by a scanner with higher magnetic field strength, a coil with a larger number of channels, and a longer examination time. Jackowski et al. [3] did use a 3-T scanner but did not investigate the diagnostic value in terms of sensitivity and specificity. Their studies focused on comparing the appearance of MI on postmortem MRI with the defined age stage at autopsy.

The aim of the present study is to evaluate the diagnostic value for detecting MI on postmortem MRI in terms of sensitivity and specificity based on T_1_-weighted and T_2_-weighted images acquired with a 3-T scanner and a 16-channel array coil at a scan duration of 10 to 20 min per sequence. An additional aim of this study is to assign the appearance of MI on postmortem MRI to the age stage as defined by Jackowski et al. [3] and compare this MRI-based age stage with the age stage determined in conventional autopsy.

## Material and methods

### Study population

This study included 88 adult decedents (male: *n* = 69), who underwent postmortem cardiac MRI between 2013 and 2019 because of suspected ischemic heart disease before conventional autopsy. The postmortem interval (PMI) between time of death (estimated maximum value) and postmortem MRI was less than 6 days in all studies (median value: 20 h, range: 2–136 h). Conventional autopsy was performed less than 24 h after MRI examination in each case. All examinations were performed on behalf of the legal case clarification within the framework of the forensic medical service. In a waiver letter, the responsible ethics committee states that it has no objections to the study being conducted.

### Gold standard

In this study, the results of autopsy and, if performed, histology are considered the gold standard. The autopsies were performed by rotating teams consisting of a senior forensic pathologist, a resident, and a technical autopsy assistant. Relevant information on the presence or absence of MI, as well as the age stage of MI, was obtained from autopsy reports. As part of the routine case workup, the forensic pathologists were informed of previous radiologic examinations and their results. Histological examination was performed in cases where the forensic pathologist in charge considered it appropriate in the context of the case clarification and the competent authorities gave their consent. The detection of an MI and its age estimation were based on the diagnostic parameters described in *Robbins Pathologic Basis of Disease* [8] (Table 1), which are applied at the Institute of Forensic Medicine.

**Table 1:** Diagnostic parameters according to *Robbins Pathologic Basis of Disease* [8] (histology and autopsy) and according to Jackowski et al. [3] (T_2_ MRI and T_1_ MRI)

**Time**	**Histology**	**Autopsy**	**T**_**2**_** MRI**	**T**_**1**_** MRI**	**Age stage**
1–2 h	• Waviness of fibers at border		• Cloudy hypointense area		Peracute
4–12 h	• Beginning coagulation necrosis • Edema • Hemorrhage • Beginning neutrophilic infiltrate	• Possible slight pallor accompanied by a detected occlusion of the corresponding coronary artery*	• Hypointense area		
18–24 h	• Continuing coagulation necrosis • Pallor (pyknosis of nuclei, shrunken eosinophilic cytoplasm) • Marginal contraction band necrosis	• Pallor	• Hypointense area surrounded by hyperintense zone** (contrast between the two regions increases with time)	• Isointense (or hyperintense) area surrounded by hypointense zone**	Acute
24–72 h	• Total coagulative necrosis with loss of nuclei and striations • Heavy interstitial infiltrate of neutrophils	• Yellowish discoloration* • Sometimes hyperemia			
3–7 days	• Beginning disintegration of dead myofibers and resorption of sarcoplasm by macrophages • Onset of marginal fibrovascular response	• Hyperemic border • Central yellow–brown softening			
10 days	• Well-developed phagocytosis • Prominent granulation tissue with fibrovascular reaction in margins	• Maximally yellow and soft vascularized margins • Red-brown and depressed • Grayish discoloration*	• Hyperintense area • Sometimes minor zones of chronification		Subacute
7th week	• Scar tissue*	• Scarring complete	• Hypointense area (myocardial scars)	• Hypointense area (myocardial scars)	Chronic

^*^In-house supplemented and applied diagnostic parameters based on own experience; ^**^According to Jackowski et al. [3], the T_2_ hyperintense/T_1_ hypointense surrounding zone corresponding to edema is visible after about 6 h

### MRI protocol

Cardiac MRI was performed using a 3-T scanner (*Achieva 3.0 TX, Philips Healthcare, Best, the Netherlands*) and a 16-channel SENSE torso coil. The scan protocol included a T_2_-weighted turbo spin echo (TSE) sequence in four-chamber view (TR: 3030 ms | TE: 100 ms | matrix: 884 × 663 mm | averages: 4) and in short axis view (TR: 4040 ms | TE: 100 ms | matrix: 884 × 663 mm | averages: 3); a T_2_-weighted sequence with spectral presaturation inversion recovery (SPIR) in short axis view (TR: 2010 ms | TE: 60 ms | matrix: 776 × 583 mm | averages: 4); and a T_1_-weighted TSE sequence in short axis view (TR: 674 ms | TE: 10 ms | matrix: 1032 × 775 mm | averages: 4). The number of slices was adapted to the size of the heart and thus the scan time of the individual sequences varied between 10 and 20 min.

### MRI evaluation

Two radiology residents with 3 and 2 years of experience in diagnostic radiology, respectively, and 1 year of specialization in cardiac MRI each screened all collected T_1_-weighted and T_2_-weighted images for the presence or absence of MI without knowing the autopsy results. MI was diagnosed on the basis of hyperintense or hypointense areas in the myocardium. The two rater were not employed at the Institute of Forensic Medicine and had no previous contact with any of the cases within the scope of the forensic medical service.

In addition, a radiologist from the Institute of Forensic Medicine with 5 years of experience in postmortem forensic radiology evaluated the T_1_- and T_2_-weighted images of all cases in which MI was detected at autopsy (true positives) and those in which MRI indicated MI that was not detected at autopsy (false positives) to verify the visual appearance of the infarct area on the images. In this evaluation, the infarct area was divided into two zones, a central infarct zone and a surrounding zone, and the appearance of each zone was classified as hypointense, isointense, or hyperintense on the T_1_-weighted and T_2_-weighted images. Based on the appearance, a classification of the age stage was made according to Jackowski et al. [3] (Table 1). Finally, any MI that was false positive was also reviewed for possible peracute MI, which was classified as such if autopsy revealed occlusion of the corresponding coronary artery.

### Statistical analysis

The *R* programming language (*RStudio, Boston, MA, USA*) was used to perform the statistical analyses. The kappa statistic was used to test interrater reliability between the two radiology residents with a confidence level of 0.95. According to Landis and Koch, a kappa (*κ*) of 0.00–0.20 was considered poor, of 0.21–0.40 fair, of 0.41–0.60 moderate, of 0.61–0.80 substantial, and of 0.81–1.00 almost perfect agreement [9]. To assess the diagnostic value of postmortem MRI for the detection of MI in decedents, the sensitivity and specificity were calculated for rater 1 and rater 2 based on the assessment of the two individual raters, with conventional autopsy considered the gold standard. A Clopper-Pearson 95% confidence interval (CI_95%_) was calculated for sensitivity and specificity.

## Results

Overall, conventional autopsy revealed MI in 34/88 decedents who underwent MRI. Of these 34 MI, rater 1 and rater 2 each identified 18 MI on the MRI data. This results in a sensitivity of 52.94% (CI_95%_: 35.13–70.22%) for both raters. The specificity was 85.19% (CI_95%_: 72.88–93.38%) for rater 1 and 92.59% (CI_95%_: 82.11–97.94%) for rater 2. With a *κ* = 0.78 (CI_95%_: 0.67–0.90), the calculation of interrater reliability calculation yielded an substantial agreement between rater 1 and rater 2.

Of the 88 autopsies (including 34 with additional histologic examination), MI was detected in 34 autopsies (including 11 with additional histologic examination). According to the autopsy reports, the age stage of MI was peracute in 7/34 cases, acute in 25/34 cases, and chronic in 2/34 cases. In none of the cases was MI explicitly classified as subacute. In individual cases of acute MI, a predamaged heart was documented with isolated chronic infarct areas. These additional chronic MI were not included in the sensitivity and specificity calculations because the inclusion of these would distort the perception of the sensitivity and specificity of postmortem MRI for the detection of MI. Comparison of the MRI data with the autopsy reports by the third rater allowed the discovery of MI that was not detected by the initial two raters. The visual appearance of the infarct area on MRI in terms of hypointensity, isointensity, and hyperintensity and the corresponding age stage are shown in Table 2.

**Table 2:** Comparison of cardiac findings in postmortem MRI and at autopsy

**No**	**R1**	**R2**	**Age stage** (autopsy)	**Age stage** (MRI)	**T**_**2**_** MRI** (central zone)	**T**_**2**_** MRI** (surrounding zone)	**T**_**1**_** MRI** (central zone)	**T**_**1**_** MRI** (surrounding zone)
1	TP	TP	Peracute*	Peracute	↓	↔	↔	↔
2	TP	TP	Peracute	Peracute	↓	↔	**↔**	↑
3	FN	FN	Peracute*	Peracute	↓	↔	↔	↔
4	FN	FN	Peracute*	Peracute	↓	↔	↔	↔
5	FN	FN	Peracute	Peracute	↓	↔	↑	↑
6	FN	FN	Peracute	*n.d*	*n.d*	*n.d*	*n.d*	*n.d*
7	FN	FN	Peracute*	*n.d*	*n.d*	*n.d*	*n.d*	*n.d*
8	TP	TP	Acute	Peracute	↓	↔	↔	↔
9	FN	FN	Acute	Peracute	↓	↔	↔	↔
10	FN	FN	Acute	Peracute	↓	↔	↔	↔
11	FN	FN	Acute*	Peracute	↓	↔	↔	↔
12	TP	TP	Acute*	Acute	↓	↑	↔	↔
13	TP	TP	Acute	Acute	↓	↑	↔	↔
14	TP	FN	Acute	Acute	↓	↑	↔	↔
15	TP	TP	Acute	Acute	↓	↑	↑	↔
16	TP	TP	Acute	Acute	↓	↑	↔	↔
17	TP	TP	Acute*	Acute	↓	↑	↔	↔
18	TP	TP	Acute	Acute	↓	↑	↑	↔
19	TP	TP	Acute	Acute	↓	↑	↑	↔
20	TP	TP	Acute*	Acute	↓	↑	↓	↔
21	FN	FN	Acute	Acute	↓	↑	↔	↔
22	FN	FN	Acute	Acute	↓	↑	↔	↔
23	FN	FN	Acute	Acute	↓	↑	↑	↔
24	FN	TP	Acute*	Subacute	↑	↔	↔	↔
25	TP	FN	Acute*	Subacute	↑	↓	↔	↔
26	TP	TP	Acute	Subacute	↑	↔	↔	↔
27	TP	TP	Acute	Subacute	↑	↔	↔	↔
28	TP	TP	Acute	Subacute	↑	↔	↔	↔
29	TP	TP	Acute	Subacute	↑	↔	↔	↔
30	FN	FN	Acute*	Subacute	↑	↔	↔	↔
31	FN	FN	Acute	Subacute	↑	↔	↔	↔
32	FN	FN	Acute	Subacute	↑	↔	↔	↔
33	TP	TP	Chronic	Chronic	↓	↔	↔	↔
34	FN	TP	Chronic	Chronic	↓	↑	↔	↔

*R1* rater 1, *R2* rater 2, *TP* true positive, *FN* false negative, *n.d.* not detected, ↓ hypointense, ↔ isointense, ↑ hyperintense, *additional histological examination

**Fig. 1:**
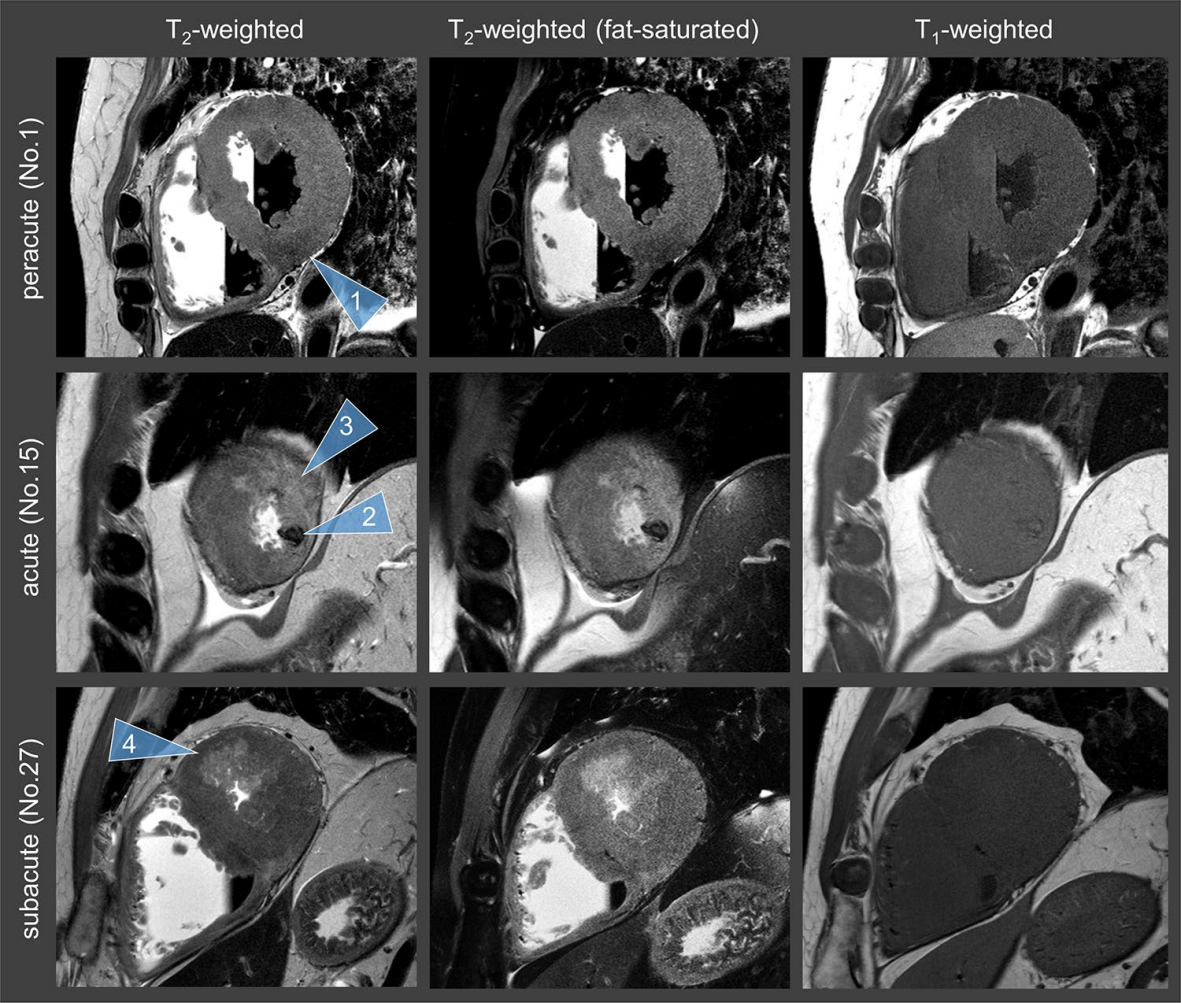
T_2_-weighted images (first column), “fat-saturated” T_2_ images (middle column), and T_1_-weighted images (right column) of peracute MI (first row), acute MI (second row), and subacute MI (third row). The term “fat-saturated” was placed in quotation marks because no temperature correction of the inversion time was made for adequate fat suppression. The indicated numbers (No.) correspond to those in the table. On the T_2_-weighted images, a peracute as well as an acute MI appears dark (arrowheads 1 and 2). An acute MI usually shows edema in the outer zone, which is bright on T_2_-weighted images (arrowhead 3). A bright infarct zone on T_2_-weighted images is associated with a subacute MI. T_1_-weighted images show little contrast between affected infarct area and unaffected tissue

Only in two cases, MI documented as peracute at autopsy could not be detected on T_1_-weighted and T_2_-weighted images. Four of the MI classified as acute according to autopsy were classified as peracute based on MRI data. In addition, nine cases that were classified as acute at autopsy appear as subacute in the MRI data. MI detection and age stage classification were largely based on the T_2_-weighted images. On the T_1_-weighted images, the infarct areas were not detectable in most cases and were perceived to be isointense (Fig. 1).

In two cases that were scored as false positives, the third rater also clearly detected an infarct area on MRI. Therefore, postmortem MRI examination suggested a peracute MI that was not identified at autopsy. However, because vascular occlusion was identified at autopsy in each of these two cases, an incipient (peracute) MI was considered plausible.

## Discussion

The results of this study show that the diagnostic value of T_1_- and T_2_-weighted 3-T MRI for the detection of postmortem MI has high specificity (approximately 85–93%) but low sensitivity (approximately 53%). Only in direct comparison with the autopsy reports and the information on the localization of the infarct area, largely all MI identified in the autopsy could also be recognized as such on the MRI data. However, two cases were also considered to have possible peracute myocardial infarction that was not detected at autopsy.

In this study, four cases were diagnosed as peracute based on their appearance on MRI, whereas they were recorded as acute at autopsy. The classification in MRI refers to the fact that in the T_2_-weighted images, no edema, i.e., a bright (hyperintense) surrounding of the dark (hypointense) infarct area, was seen, which according to Jackowski et al. [3] allows to distinguish between peracute and acute [3]. However, it cannot be excluded that in this MI, simply no edema has formed at all. There are also different opinions about how early edema would be seen on MRI. In 2005, Shiotani et al. [10] presented a case with increased signal intensity in the myocardium on T_2_-weighted imaging associated with acute MI but without corresponding macroscopic and histologic findings. The authors considered this to be edema due to coronary artery occlusion, which is thought to develop in the first 30 min and would then already be seen on water-sensitive MRI, referring to an MRI study on dog hearts from 1980 [11]. In a more recent MRI study of porcine hearts, Ruder et al. [12] also suggest that edema develops rapidly after coronary artery occlusion, with results as early as 3 h. Regardless of how early edema develops and would be detectable on MRI, there is agreement that a postmortem MRI examination can indicate very recent MI, earlier than would be apparent macroscopically at autopsy or even microscopically at histology. Returning to the results of the present study, the surrounding zone that was bright on T_2_- weighted images did not appear dark on T_1_-weighted images as expected, but this may be related to postmortem temperature effects. In general, T_1_-weighted images were not supportive in classifying age stage. In nine cases, T_2_-weighted images showed a bright infarct area, which is described as an indication of the subacute age stage [3]. The autopsy largely indicated an acute age stage but in many cases did not differentiate more precisely between acute and subacute. This is probably due to the fact that no histological clarification was carried out in most of these cases. Accurate determination of the age stage of an infarct area based on macroscopic findings alone without additional histologic examination can be difficult to unfeasible. Therefore, in these cases where no histological examination was performed, the gold standard in terms of age estimation may be questioned. However, there is also a discrepancy between gold standard and postmortem MRI in terms of acute versus subacute age stage in three cases in which histological examination was performed. MRI imaging showed regional signal differences in myocardial tissue attributable to tissue changes in these regions. With regard to the classification of subacute MI, it appeared bright on T_2_-weighted images on postmortem MRI, whereas peracute, acute, and chronic MI were dark. This clearly suggests a change in the tissue, which is largely attributable to the age stage of the infarct area [3]. It can be concluded that postmortem MRI as an adjunct to macroscopic findings (i.e., without additional histologic examination) may provide a more accurate classification of age stage. However, with regard to hypointense areas in the myocardium, which are classified as peracute MI because of the absence of hyperintense surrounding zones due to not yet formed edema, it is unclear to what extent agonal ischemic changes may cause such hypointense areas. The mechanism of death, for example in hanging or carbon monoxide intoxication, can cause a gradual decrease in circulatory and respiratory activity that may eventually lead to agonal ischemic changes in the myocardium [13]. In immunohistochemical detection of sudden myocardial infarction, one is aware of such “agonal artifacts” [14]. However, at times, it is still unclear whether and to what extent such “agonal artifacts” are visible on postmortem MRI.

In the two cases in which a possible peracute MI was detected on postmortem MRI but was not detected at autopsy, no myocardial findings were detected at autopsy but occlusion of the corresponding coronary artery was noted. In such cases, MRI may help to determine the region from which samples could be obtained for further investigation. Sampling could be performed minimally invasively by image-guided biopsy [6, 15]. This provides the opportunity to evaluate peracute infarcts, which are difficult to detect macroscopically, for detectability by MRI or after sampling by other investigative techniques. However, it is also worth noting the general problems and challenges of image-guided postmortem biopsy, such as mediocre predictive values and needle placement accuracy [16, 17]. Nevertheless, T_2_-weighted images do provide distinguishing features that allow detection of the infarct area and determination of the age stage, making noninvasive postmortem MRI a valuable tool for research. However, due to its low sensitivity, the MRI protocol used is less suitable for forensic medical service.

With regard to the sensitivity and specificity of postmortem MRI compared with autopsy as the gold standard, it is important to reiterate that the radiology residents (rater 1 and rater 2) were blinded to the detailed case circumstances, forensic reports, autopsy reports, and initial MRI reports when evaluating the MRI data in this retrospective study, whereas the forensic pathologists at autopsy were not. This results in a certain bias towards autopsy, which is not decisive for the pure assessment of postmortem MRI with regard to the detection of MI in this study, but which has to be kept in mind when comparing postmortem MRI and autopsy directly. In the present study, a direct comparison is largely omitted. Notwithstanding this, the results on sensitivity and specificity of T_1_-weighted and T_2_-weighted imaging for the detection of MI are similar to those obtained by Wagensveld et al. [6] using a 1.5-T scanner. Therefore, it is assumed that the field strength of the scanner, the coil, and the scan time does not have a decisive influence on the diagnostic value. However, T_1_-weighted and T_2_-weighted images used are optimized for the living patient and have not been adapted for postmortem studies. In addition to postmortem influences in the context of tissue decomposition and autolysis, another factor influencing image contrast is the change in temperature. Relaxation times are temperature dependent [18]. Although the temperature effect on T_2_ relaxation is considered relatively small compared with T_1_ relaxation, temperature correction of postmortem MRI data should be sought for T_1_-weighted and T_2_-weighted imaging. For selective tissue suppression, for example, the corresponding inversion time must be known, i.e., one must know how long the longitudinal magnetization of the corresponding tissue, e.g., fat, takes until it approaches zero after applying a 180° inversion pulse. The inversion time can vary considerably postmortem as it depends on T_1_, which, in turn, depends strongly on temperature. Thus, the T_2_-weighted SPIR sequence did not achieve the desired effect for fat suppression on postmortem imaging in the present study. Nevertheless, the sequence proved to be advantageous for the detection of edema compared to the pure T_2_-weighted sequence. In general, quantitative T_1_ and T_2_ may be helpful for MI detection and age stage determination, as shown by Zech et al. [19].

Apart from the temperature effects, the low sensitivity for the detection of MI on postmortem MRI achieved in the present study and in that of Wagensveld et al. [6] may be increased by other MRI techniques. For example, in three cases studied, Crooijmans et al. [20] showed that magnetic transfer (MT) imaging can correspond to histological results. The application of an MT pulse saturates the protons bound to macromolecules, and these saturated protons exchange with unsaturated protons of free water, reducing the free water signal. The difference in the magnitude of the free water signal between an experiment with and without such an MT pulse, called the magnetic transfer ratio (MTR), is proportional to the fraction of macromolecules. Typically, such an MT pulse is an off-resonance radiofrequency (RF) pulse, but this effect can also be achieved by an on-resonance RF pulse, where a reduction in RF power deposition results in a reduction in the MT effect. The authors used a steady state free precession (SSFP) free induction decay (SSFP-FID) sequence with a short MT-weighted pulse of 310 μs and with an elongated pulse of 2100 μs to reduce the MT effect in the images. According to Crooijmans et al. [20], increased MTR may indicate fibrous tissue, as this is associated with an increased fraction of macromolecules, while edema-induced tissue swelling decreases the MTR, as this is associated with a decreased fraction of macromolecules [20]. Compared with T_2_-weighted images, MTR maps may provide more sensitive discrimination between unaffected myocardium, acute MI, and chronic MI. However, to our knowledge, this technique has not been used further for postmortem MI detection and it needs evaluation in an adequate study population.

A limitation of the present study is that histological examinations were not performed in all decedents who had MI at autopsy, and therefore, the estimate of the age stage at autopsy could not be additionally confirmed. However, this corresponds to the actual case clarification in forensic medicine, since an additional histological examination must also be ordered and financially remunerated. Another limitation of this study, which has already been discussed, is the fact that the forensic pathologist was not blinded to additional information, including the previously performed MRI examination, whereas rater 1 and rater 2 were blinded to additional information in the retrospective evaluation of the MRI data. Regarding the MRI data, a limitation of this study is that additional temperature measurements were not performed and thus the influence of temperature could not be taken into account.

In conclusion, the appearance of MI on postmortem MRI allows classification of age stage, but the low sensitivity implies the need for further additional MRI techniques to increase sensitivity and thus diagnostic value. However, even an increase in sensitivity for MI detection only improves the diagnostic value with respect to MI, and the many other pathologies that can be answered by conventional autopsy are far from being evaluated on postmortem MRI. This means that postmortem MRI is still far from adding a real value to the forensic medical service, especially considering that the effort for an MRI examination, which must be performed externally if there is no in-house scanner at the institute, is very high. However, the scientific value of postmortem MRI is given, because this noninvasive examination technique allows to detect infarct areas and, if necessary, to classify them regarding their age in relation to their MRI appearance. Especially with regard to the localization of peracute MI and the targeted sampling for further investigations, postmortem MRI shows potential scientific value.

## Key points

1. The sensitivity and specificity of T_1_- and T_2_-weighted MRI for postmortem detection of myocardial infarction with a 3-T scanner are similar to previous results with a 1.5-T scanner.
2. The low sensitivity of T_1_- and T_2_-weighted MRI for detecting postmortem myocardial infarction diminishes its value to the forensic medical service.
3. Postmortem cardiac MRI allows detection of very recent (peracute) myocardial infarction.
4. The scientific value of postmortem cardiac MRI arises from the noninvasive nature of this examination technique and the ability to classify the age stage of myocardial infarction by hyperintense, isointense, and hypointense areas in the myocardium.

### Supplementary Information

Below is the link to the electronic supplementary material.

Supplementary file1 (PDF 434 KB)

## Data Availability

The data analyzed in this study were generated in the context of forensic medical case clarification and thus cannot be made generally available. However, a request can be made to the corresponding author and will be assessed individually via the Institute of Forensic Medicine.
