# Supplementary material for: Diagnostic value of T1- and T2-weighted 3-Tesla MRI for postmortem detection and age stage classification of myocardial infarction
Source: Forensic Sci Med Pathol. 2023 Mar 2;20(1):14–22. doi: 10.1007/s12024-023-00592-8 (PMC10944381; doi:10.1007/s12024-023-00592-8)
Supplement: Supplementary file 1 — Supplementary file1 (PDF 434 KB) [file 12024_2023_592_MOESM1_ESM.pdf]

Supplementary material: Study population

(Diagnostic value of T1- and T2-weighted 3 Tesla MRI for postmortem detection and age stage classification of myocardial infarction)

To test for correlation between myocardial infarction (MI) detected at autopsy -*dependent variable*- and sex, age, heartweight, body mass index (BMI), or postmortem interval (PMI) -*independent variables*-, logistic regression analysis was used, and in case of a significant correlation the odds ratio was calculated. The chi-square test (omnibus test) was used to test whether the model as a whole was significant, and the Nagelkerke pseudo-*R*-squared (R^2_N) was calculated to evaluate the goodness of fit of the logistic regression model. A p-value of <0.05 was considered significant.

In the study group, the median value (range) of the age was 51.5 years (31 - 91 years), of the BMI was 25.9 kg/m^2 (15.0 - 37.4 kg/m^2), and of the heart weights was 425 g (250 - 770 g) (*Fig.1*). Age was found to be a significant predictor for the detection of MI (p <0.033). The logistic regression model was significant (p <0.029, R^2_N = 0.719). If the age increases by one year, the odds for MI are 1.03 times higher. Sex, heart weight, BMI, and PMI were not significant factors.

Figure: Descriptive data from the study group on age, heart weight, BMI, and PMI.

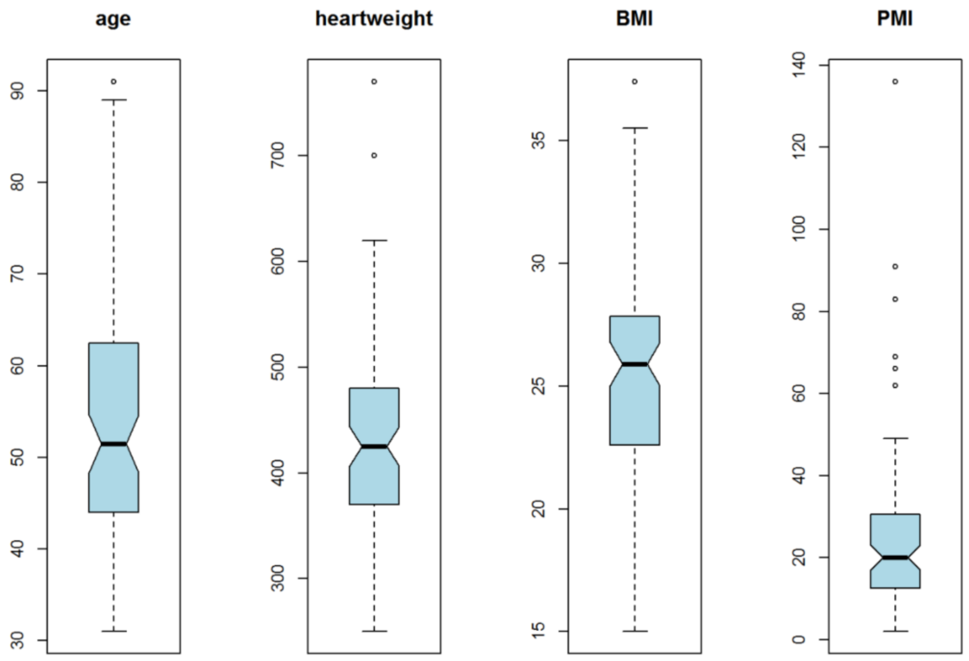

The study population showed a significant correlation between age and the presence of MI. This result is not unexpected, as the prevalence of MI increases with age [1]. The present study does not further address this additional outcome because the study objective and study population were not designed for this evaluation.

1. Yazdanyar A, Newman AB. The Burden of Cardiovascular Disease in the Elderly: Morbidity, Mortality, and Costs. Clinics in Geriatric Medicine. Elsevier; 2009;25:563–77.

Table: Details of the individual cases

no.	M = male, F = female	age [year]	heart weight [gram]	BMI	PMI [hour]
1	M	33	410	22	11
2	F	89	420	21.8	49
3	M	41	450	24.9	69
4	F	47	290	22.4	2
5	M	56	540	27.4	91
6	F	72	380	22.1	42
7	M	48	480	27.4	25
8	M	57	350	27.8	8
9	F	75	430	25.9	40
10	M	50	460	28.6	25
11	F	81	330	28	19
12	M	45	530	29.9	22
13	F	85	330	25	13
14	M	48	470	26.9	16
15	M	42	480	28.9	28
16	M	80	490	24.2	5
17	F	48	340	20.9	14
18	M	49	380	23.7	30
19	F	44	340	35.1	66
20	M	51	440	26.6	21
21	M	69	380	22.8	6
22	M	44	450	21.8	11
23	M	54	380	21.6	28
24	M	61	440	26.9	4
25	M	45	400	28.1	11
26	M	50	340	21.9	7
27	F	91	290	20.9	34
28	M	50	420	29.4	21
29	F	84	350	26	19
30	M	44	420	25.1	31
31	M	45	700	37.4	136
32	M	80	540	27.3	4
33	M	73	550	27.8	25
34	M	49	490	25.9	18
35	M	45	360	27.4	4
36	M	60	410	23.8	10
37	M	80	390	23.4	21
38	M	53	430	23.2	40
39	M	31	460	32.1	27
40	M	56	510	27.1	47
41	M	59	440	26	4
42	M	34	430	31.6	31
43	M	40	540	23.9	22
44	M	69	550	32.6	3
45	M	39	420	25.3	47
46	M	54	700	31.7	27
47	M	35	460	28.4	18
48	M	47	500	27.6	3
49	M	41	320	21.1	20
50	M	43	480	30.5	4
51	M	87	390	27.7	47
52	M	50	300	20.2	26
53	M	50	370	23.5	14
54	F	59	330	27.5	23
55	M	79	460	27.5	14
56	M	33	770	35.5	20
57	M	32	370	24.2	91
58	M	57	340	22.3	19
59	M	66	590	23.6	62
60	F	79	370	30.3	38
61	M	61	550	31	8
62	M	44	460	23.9	18
63	M	44	400	21.7	21
64	M	51	430	34.2	21
65	M	58	390	24.3	20
66	F	76	290	27.9	16
67	M	31	510	23.2	9
68	F	55	250	15	12
69	F	67	310	19.4	10
70	M	55	410	22.2	40
71	M	61	440	22	19
72	M	32	380	24.9	28
73	M	38	430	16.9	83
74	M	53	410	25	14
75	F	55	360	18.4	44
76	M	44	430	26.9	13
77	M	52	460	27.3	5
78	M	35	370	19.9	27
79	M	36	410	26.3	20
80	M	66	620	30.2	17
81	M	63	450	27.3	15
82	F	32	280	26.9	14
83	M	39	560	28.7	15
84	M	36	440	20.1	3
85	F	61	360	21.5	35
86	M	55	520	25.9	38
87	F	83	360	24.1	20
88	M	62	520	27.9	22
